# Author Correction: Cerebellar Purkinje cells combine sensory and motor information to predict the sensory consequences of active self-motion in macaques

**DOI:** 10.1038/s41467-024-48996-6

**Published:** 2024-06-05

**Authors:** Omid A. Zobeiri, Kathleen E. Cullen

**Affiliations:** 1 Department of Biomedical Engineering, McGill University, Montréal, QC Canada; 2 Department of Biomedical Engineering, Johns Hopkins University, Baltimore, MD USA; 3 Department of Otolaryngology-Head and Neck Surgery, Johns Hopkins University School of Medicine, Baltimore, MD USA; 4 Department of Neuroscience, Johns Hopkins University School of Medicine, Baltimore, MD USA; 5 Kavli Neuroscience Discovery Institute, Johns Hopkins University, Baltimore, MD USA

**Keywords:** Reflexes, Sensory processing

Correction to: *Nature Communications* 10.1038/s41467-024-48376-0, published online 11 May 2024

In this article the title was incorrectly given as Cerebellar Purkinje cells in male macaques combine sensory and motor information to predict the sensory consequences of active self-motion but should have been Cerebellar Purkinje cells combine sensory and motor information to predict the sensory consequences of active self-motion in macaques. The original article has been corrected.

