# Supplementary material for: Cerebellar Purkinje cells combine sensory and motor information to predict the sensory consequences of active self-motion in macaques
Source: Nat Commun. 2024 May 11;15:4003. doi: 10.1038/s41467-024-48376-0 (PMC11088633; doi:10.1038/s41467-024-48376-0)
Supplement: Supplementary file 1 — Supplementary Information [file 41467_2024_48376_MOESM1_ESM.pdf]

Supplementary Fig. 1

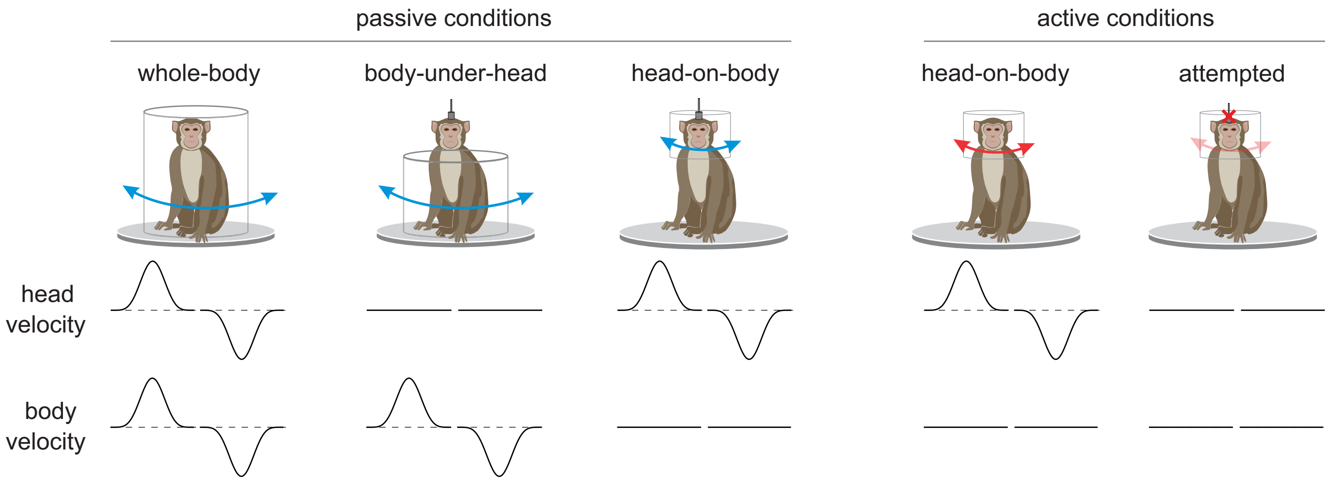

Supplementary Fig. 1 – Schematic of experimental conditions in the study. From left to right: passive whole-body rotation (vestibular), passive body-under-head rotations (neck proprioception), passive head-on-body rotation (vestibular + neck proprioception), active head-on-body rotation (vestibular + neck proprioception + neck motor command), attempted head-on-body rotation (neck motor command).

Supplementary Fig. 2

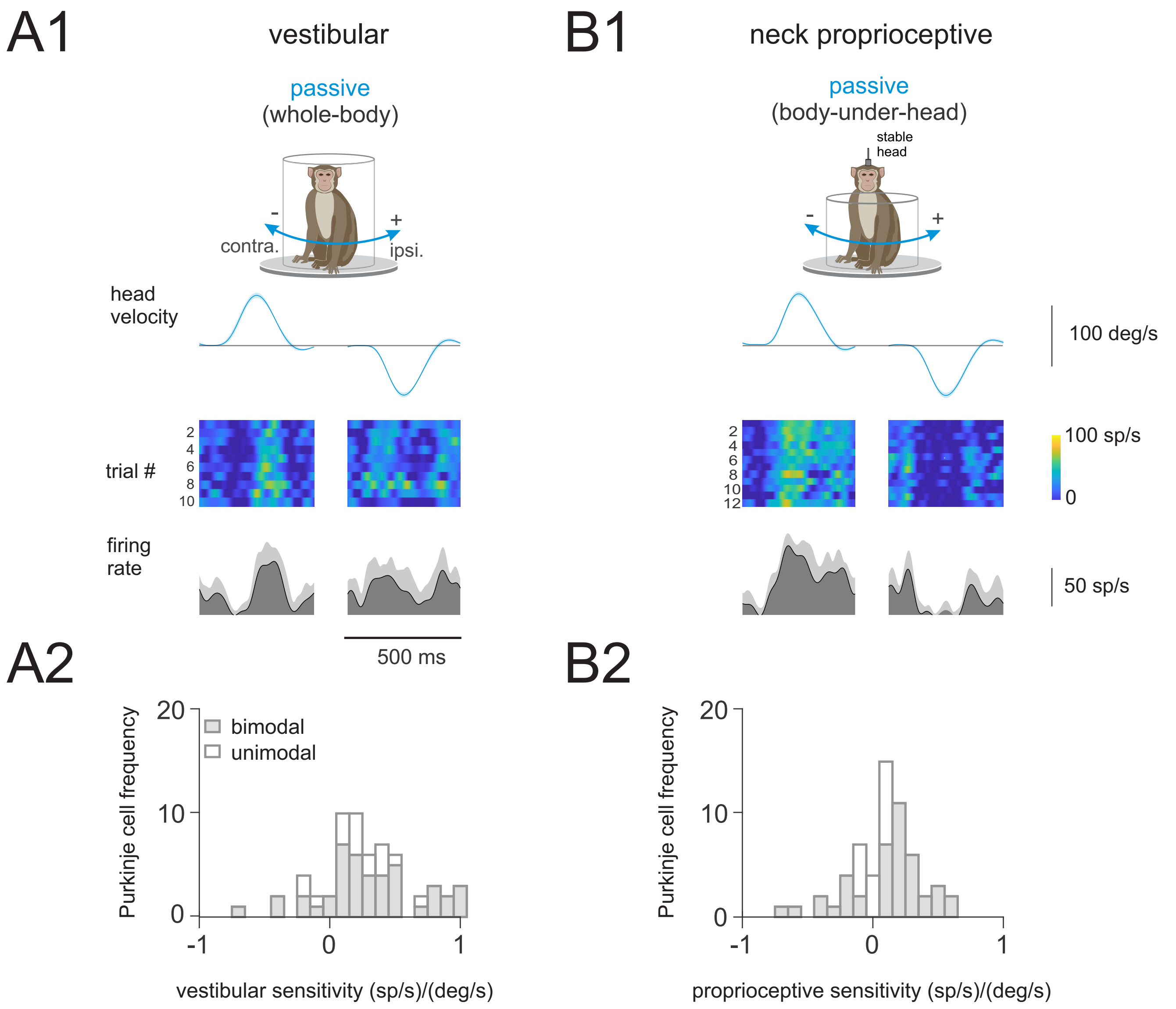

Supplementary **Fig. 2 –** Comparison of Purkinje cell responses to (A) whole-body (vestibular) and (B) body-under-head (proprioceptive) rotations in the preferred movement direction.

Supplementary Fig. 3

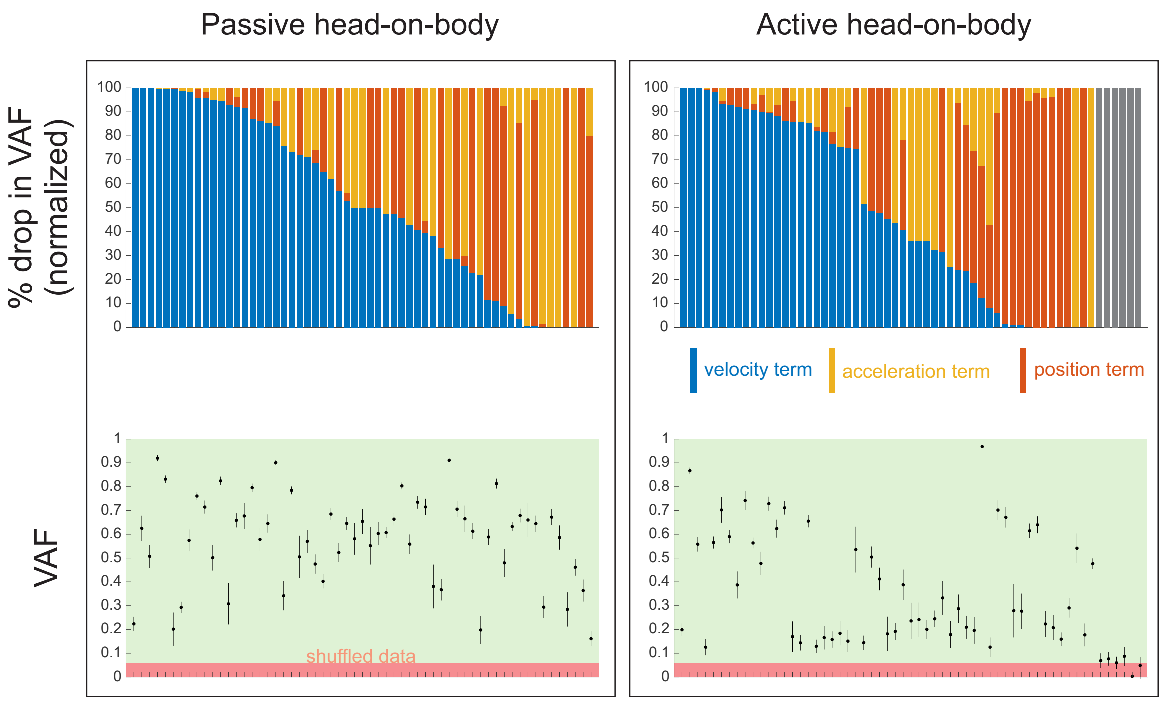

Supplementary Fig. 3 – The contribution of each kinematic term (i.e., position, velocity, acceleration) in estimating the firing rate for the passive (left) and active (right) head-on-body conditions, computed as the % drop in total variance-accounted-for (VAF) when removed from the full model. Neurons that did not significantly respond are shown as gray bars. Note, we sorted Purkinje cells based on the importance of the velocity term, since this is what is predominately encoded by their target neurons in the rostral fastigial nucleus (rFN) and vestibular nuclei.

Supplementary Fig. 4

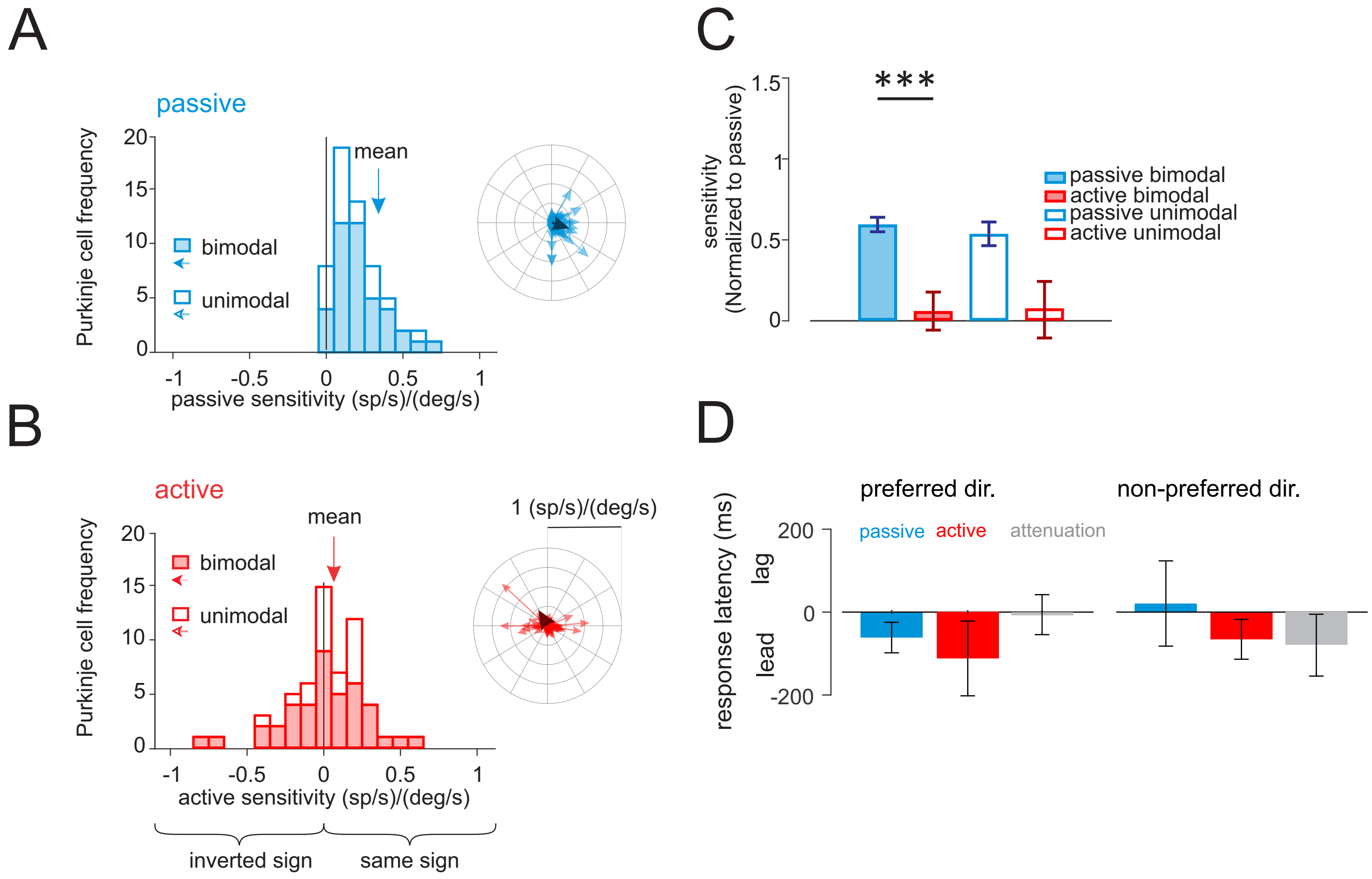

Supplementary Fig. 4 – Comparison between the Purkinje cell responses during active vs. passive head movements for non-preferred direction of movement. (A) Distribution of neural sensitivity of the bimodal (filled bars) and unimodal (open bars) Purkinje cells for passive self-motion in the preferred direction (i.e., the direction resulting in the larger increase in simple spike firing rate) (B) Same as (A) for active self-motion. Insets: polar plots where the vector length and angle represent each neuron's vestibular response sensitivity and phase, respectively. Dark arrows represent the average response. (C) Bar plots comparing the normalized sensitivity of the bimodal and unimodal Purkinje cells during passive vs. active head movements (Two-sided t-test, corrected for multiple comparasion, *** $p<0.001$). (D) the response latency of the Purkinje cells response during passive (blue) and active (red) conditions and the attenuation (i.e. passive-active, grey). Data presented as mean values +/- SEM. Two-sided ttest test did not show any differences ($p>0.5$).

Supplementary Fig. 5

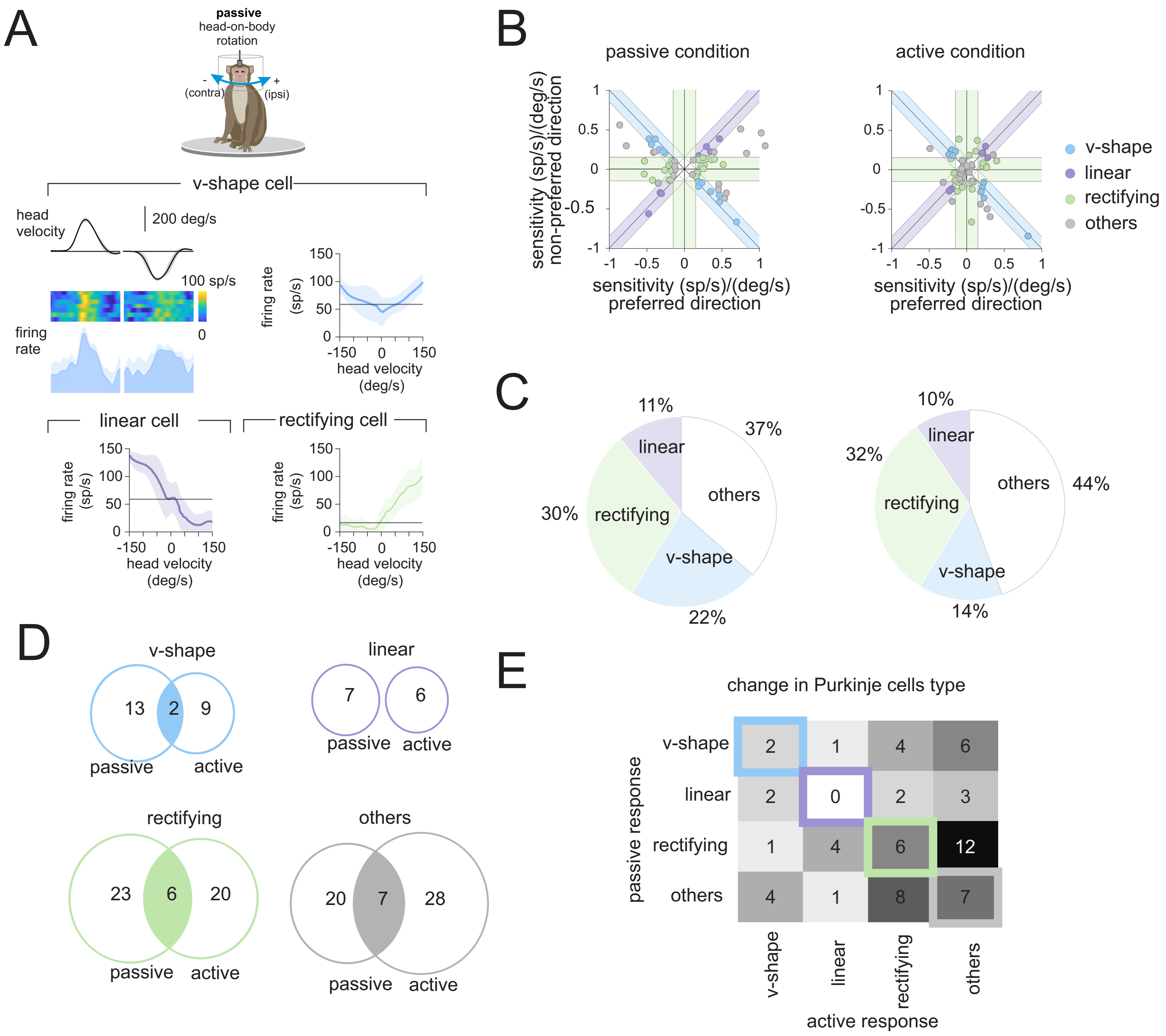

Supplementary Fig. 5 - Purkinje cells show heterogeneity in their simple spike responses during passive and active head movements. (A) The responses of the Purkinje cells during passive head-on-body motion, which could be V-shape, which demonstrated increased firing rate in both directions, linear, which demonstrated increased and decreased firing rate in the preferred and non-preferred directions, respectively, and rectifying, that demonstrated increased firing rate in the preferred direction and minimal modulation in the non-preferred direction. (B) The scatter plots that show the sensitivity of the Purkinje cells during passive (left) and active (right) head-on-body motion, which are groups as (i) V-shape, which demonstrated increased firing rate in both directions, (ii) linear, which demonstrated increased and decreased firing rate in the preferred and non-preferred directions, respectively (iii) rectifying, that demonstrated increased firing rate in the preferred direction and minimal modulation in the non-preferred direction, and (iv) others, that did not meet any of the mentioned criteria. (C) The pie charts illustrate the percentage of each category within the Purkinje cells during passive and active head-on-body movements. (D) Venn diagrams showing the number Purkinje cells that their responses were grouped as linear, V-shape, rectifying, and other during passive and active head-on-body movements and their overlap. (E) The number of Purkinje cells that were classified in pairs of groups based on their response during passive and active head-on-body movements.

Supplementary Fig. 6

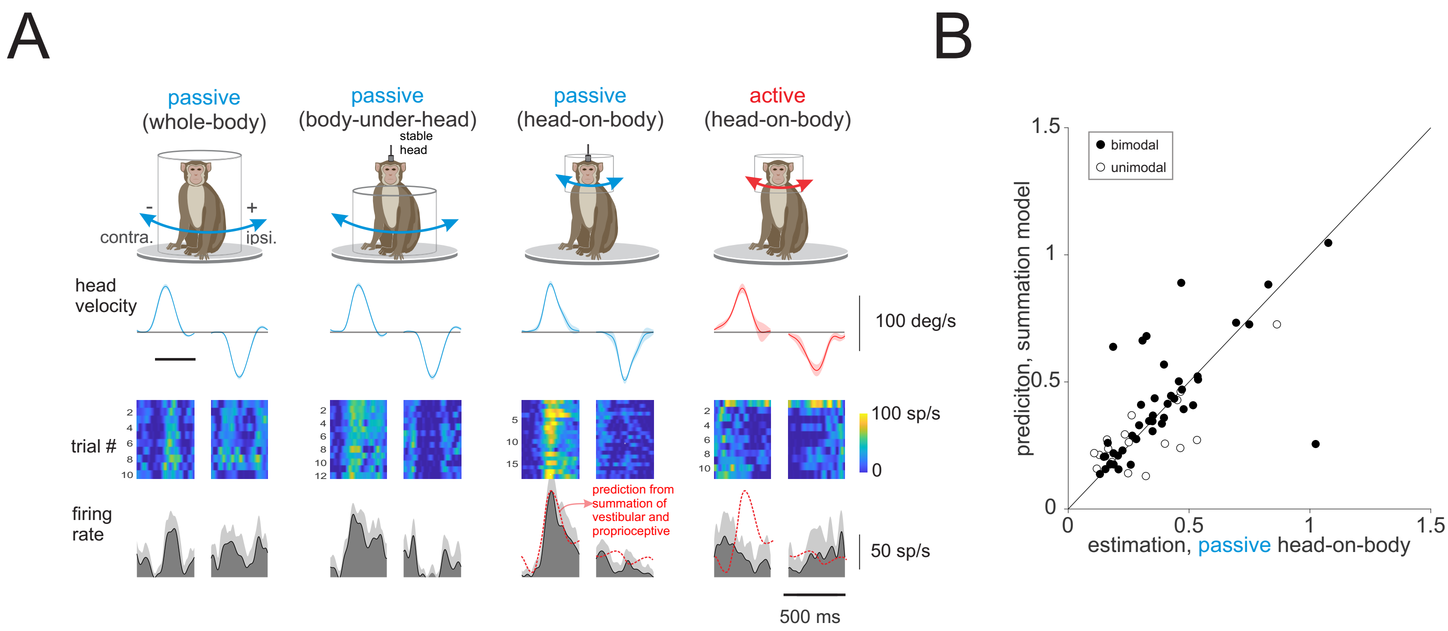

Supplementary Fig. 6 – (A) The response of an example Purkinje cells during passive whole-body (vestibular), body-under-head (proprioceptive), head-on-body (vestibular and proprioceptive), and active head-on-body (vestibular, proprioceptive, and motor command).The linear summation of neck proprioceptive and vestibular sensitivities (dashed red traces) could predict the response of the Purkinje cells during passive but not active head-on-body condition. (B) Comparison of predicted sensitivities based on summation of a given neuron's responses to vestibular and proprioceptive stimulation when applied alone and estimated during passive head-on-body rotations in the preferred movement direction. The linear summation of a given neuron's vestibular, and neck proprioceptive sensitivities well predicts sensitivity during the passive condition.

Supplementary Fig. 7

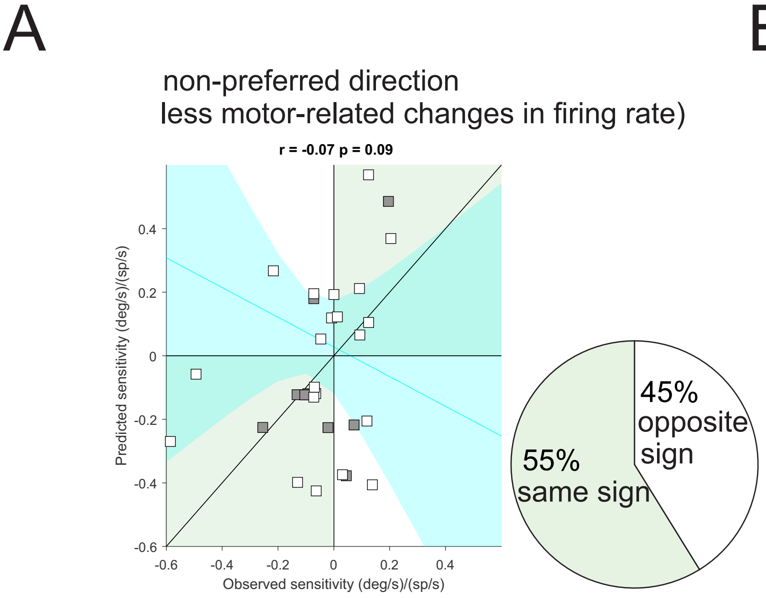

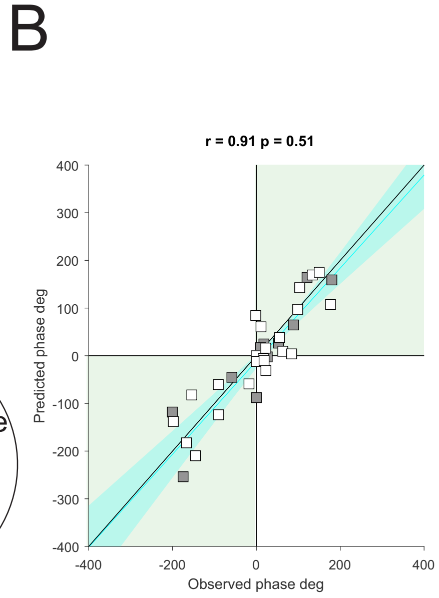

Supplementary Fig. 7 – Scatter plots comparing the observed vs. predicted gain (A) and phase (B) of the Purkinje cell's response during active head movements for the direction with the smallest change due to motor-related inputs. Blue lines and shading denote the mean ± 95% CI of linear fit.

Supplementary Fig. 8

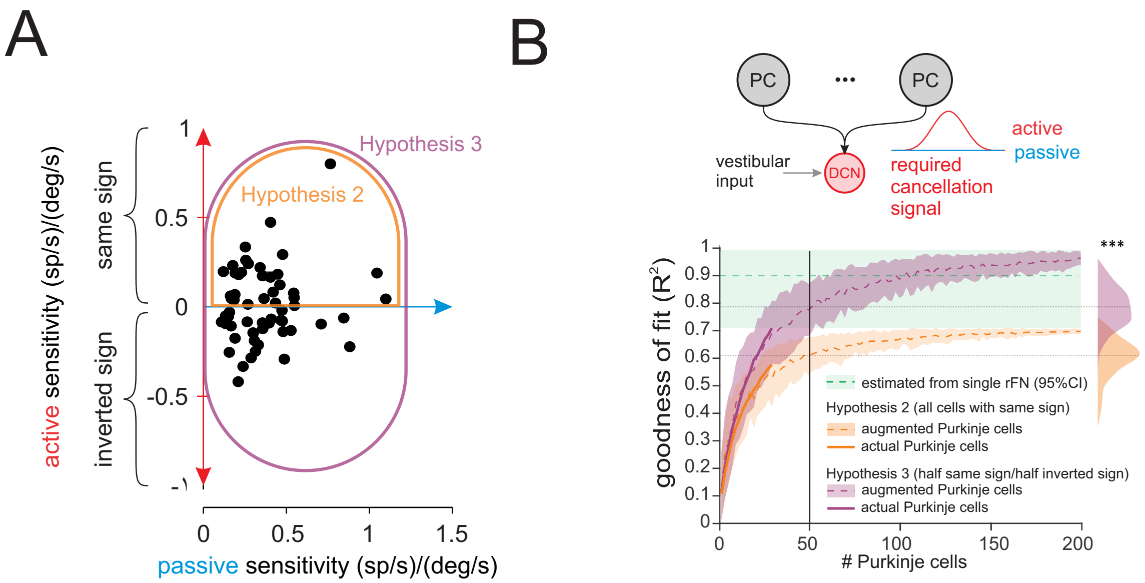

Supplementary Fig. 8 – Heterogeneity in Purkinje cell responses is required for optimal generation of the cancellation signal (A) The Purkinje cells that were used for Hypothesis 2 (orange) and Hypothesis 3 (black) (B) Comparison between the performance of the Purkinje cells (PC) population model providing cancellation signal to a deep cerebellar nuclei (DCN) neuron, based on Hypothesis 2 and Hypothesis 3. Lines and shading denote the mean ± 95% CI of linear fit.

Supplementary Fig. 9

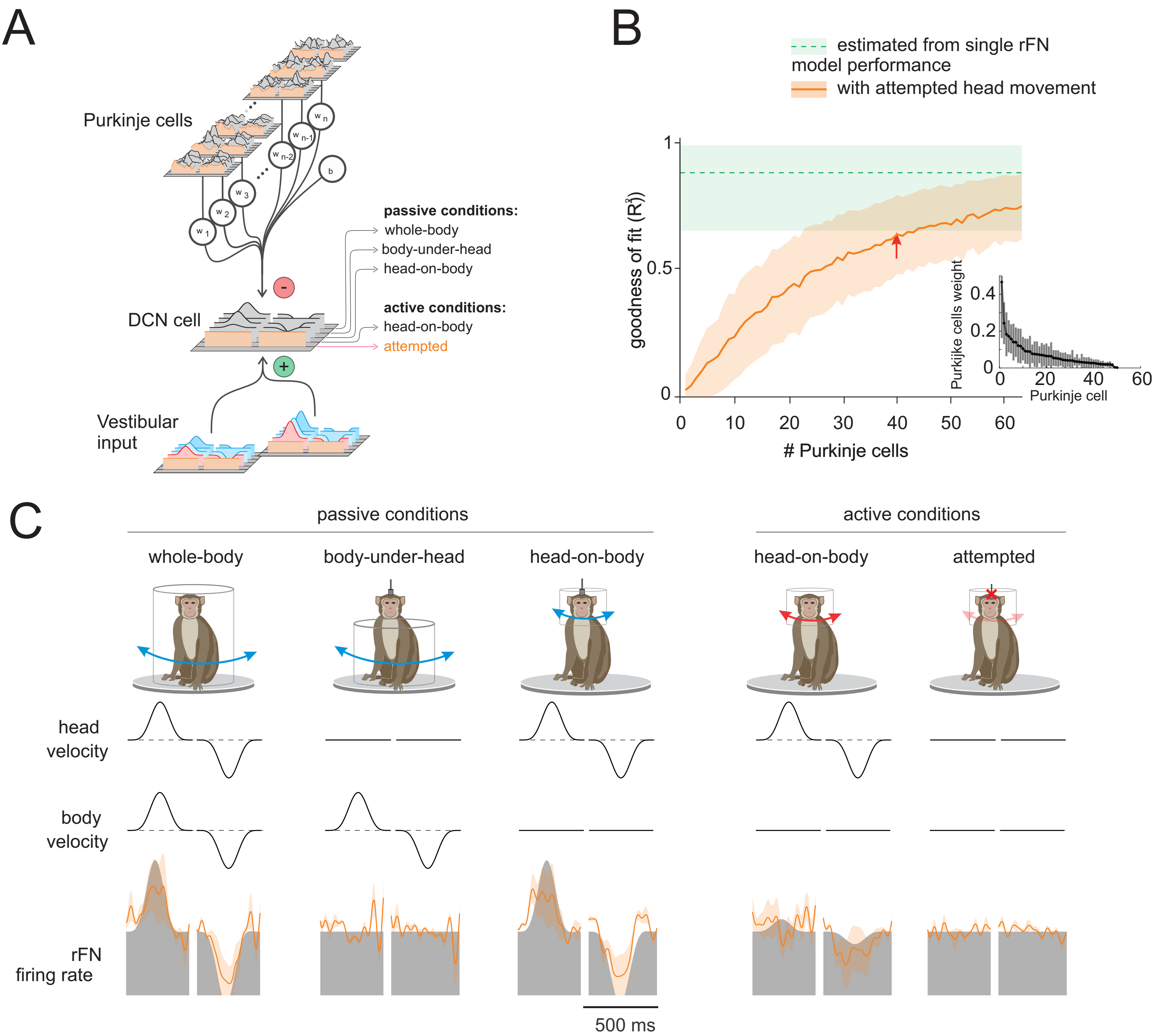

Supplementary Figure 9 – A simple linear population model of Purkinje cell integration can explain the responses of unimodal target neurons in deep cerebellar nuclei across all self-motion conditions. (**A**) Schematic of the linear summation population model used to estimate the firing rate of a target neuron in the rFN. Each Purkinje cell's weight was optimized to generate the best estimate of the average bimodal rFN neuron across conditions (Brooks and Cullen 2013). (**B**) Model performance as a function of the number of Purkinje cells. The orange curve corresponds to the model fit to simple spike firing rates of all Purkinje cells during these same four conditions as well as in simulated responses in the attempted head movement condition (**Figure 5**, see Methods). The variability estimated from a population of rFN bimodal neurons previously described by Brooks and Cullen (2009, 2013) is represented by the green shaded band. *Inset*: the distribution of computed weights for each Purkinje cell modeled during our three dynamic conditions with 40 Purkinje cells, sorted based on average weight. (**C**) Estimated model firing rates based on a population of 40 Purkinje cells superimposed on the actual average firing rate of a bimodal rostral fastigial nucleus (rFN) neuron (grey shaded region). Orange lines illustrate firing rate estimations from models that included the four conditions as well as the attempted head movement condition.

Supplementary Fig. 10

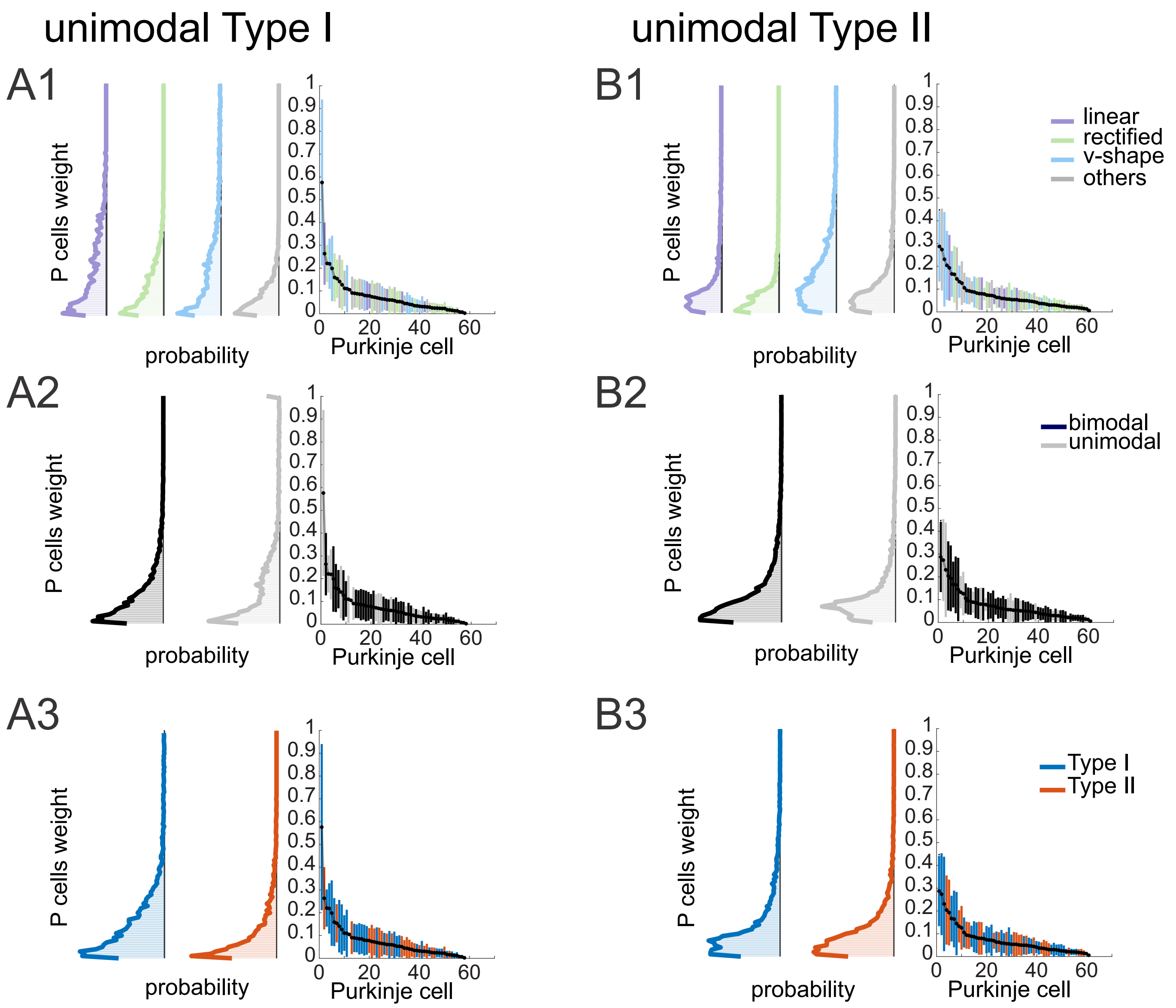

Supplementary Fig. 10 – The distribution of the weights of the inputs to the model with 40 Purkinje cells projecting to a unimodal rFN neuron. The distribution of the Purkinje cell weights in a model with 40 Purkinje cells projecting to Type I (left columns) and Type II (right column) rFN neuron that were classified as (A1, B1) linear vs. v-shaped vs. rectifying Purkinje cells, (A2, B2) bimodal vs. unimodal Purkinje cells, and (A3, B3) Type I vs. Type II Purkinje cells.

Supplementary Fig. 11

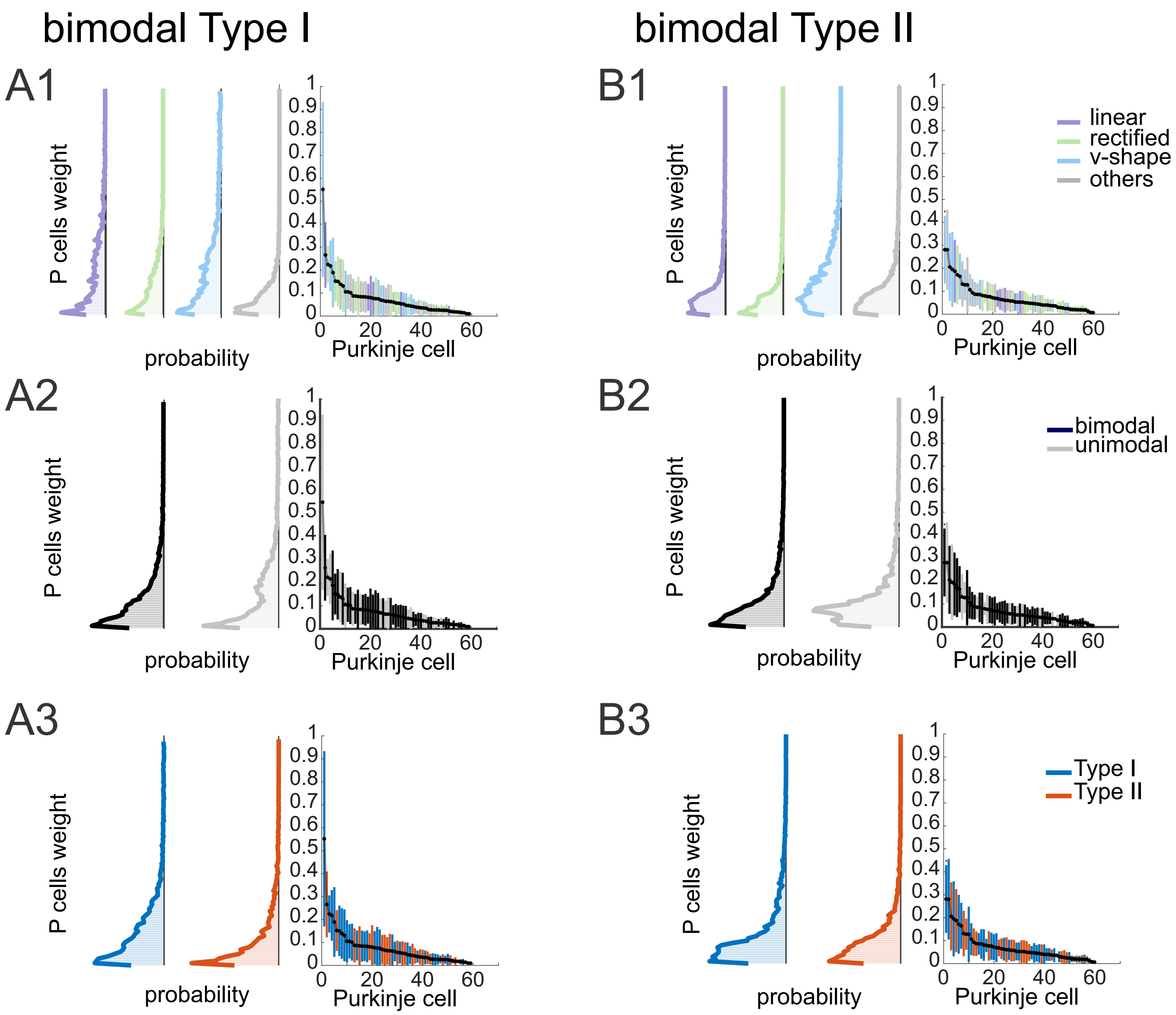

Supplementary Fig. 11 – The distribution of the weights of the inputs to the model with 40 Purkinje cells projecting to a bimodal rFN neuron. The distribution of the Purkinje cell weights in a model with 40 Purkinje cells projecting to Type I (left columns) and Type II (right column) rFN neuron that were classified as (A1, B1) linear vs. v-shaped vs. rectifying Purkinje cells, (A2, B2) bimodal vs. unimodal Purkinje cells, and (A3, B3) Type I vs. Type II Purkinje cells.

Supplementary Fig. 12

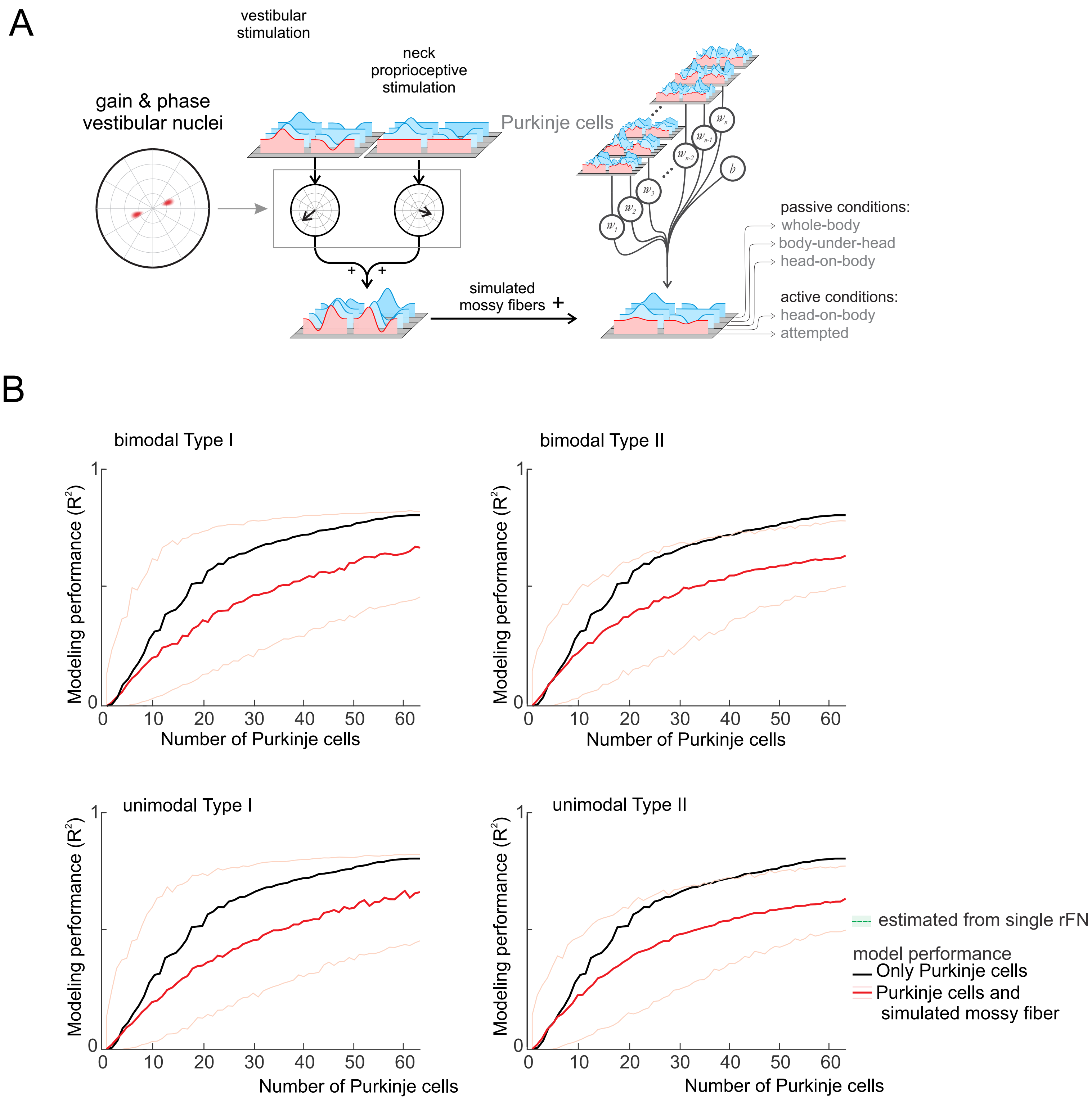

Supplementary Fig. 12 – Modeling the mossy fiber inputs to fastigial neurons. (A) Schematic of a model with a mossy fiber input that is simulated as the summation of random patterns of responses to vestibular and neck proprioceptive input. The gains and phases were randomly drawn from a distribution comparable to that previously reported in the vestibular nuclei (0.6±0.1 (sp/s)/(deg/s) and 20±5 deg, respectively; Mitchell er al. 2017). (B) The performance of the Purkinje cell population in predicting the response of the Type I (left) and Type II (right) rFN neurons in the presence (grey) and absents (red) of mossy fibers in (A). Data presented as mean values +/- 95% CI.

Supplementary Fig. 13

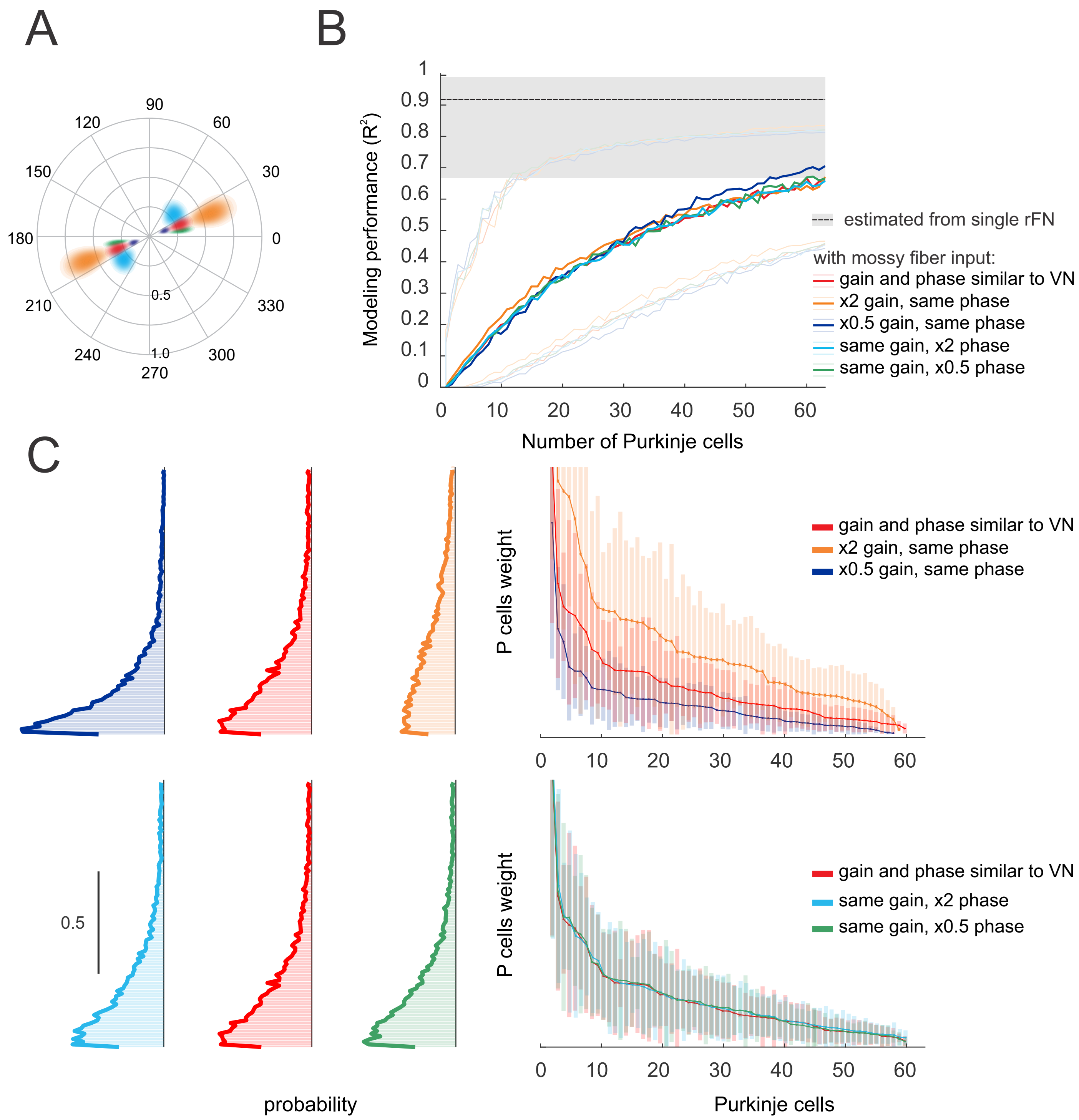

Supplementary Fig. 13 - The effect of systematically altering the distribution of gain and phase values in this simulated mossy fiber input. (A) Five different distributions of mossy fiber inputs. The reference distribution is similar to the neurons in the vestibular nuclei (red; gain: 0.6±0.1 (sp/s)/(deg/s), phase: 20±5 deg). Four other distributions were generated by systematically altering the distribution of gain and phase values for the reference distribution by i) doubling the gain (orange), ii) reducing the gain by half (dark blue), iii) doubling the phase (blue), and iv) reducing the phase by half (green). (B) The performance of a population model of Purkinje cells projecting to a Type I rFN neuron considering mossy fiber inputs with the five distributions shown in (A). The performance was similar for all distributions of mossy fibers. Data presented as mean values +/- 95% CI. (C) The distribution of the weights of the Purkinje cells in the model with 40 Purkinje cells considering mossy fiber inputs with the distributions shown in (A). Changing the gain but not the phase of mossy fiber inputs affects the weighting of Purkinje cell inputs.

Supplementary Fig. 14

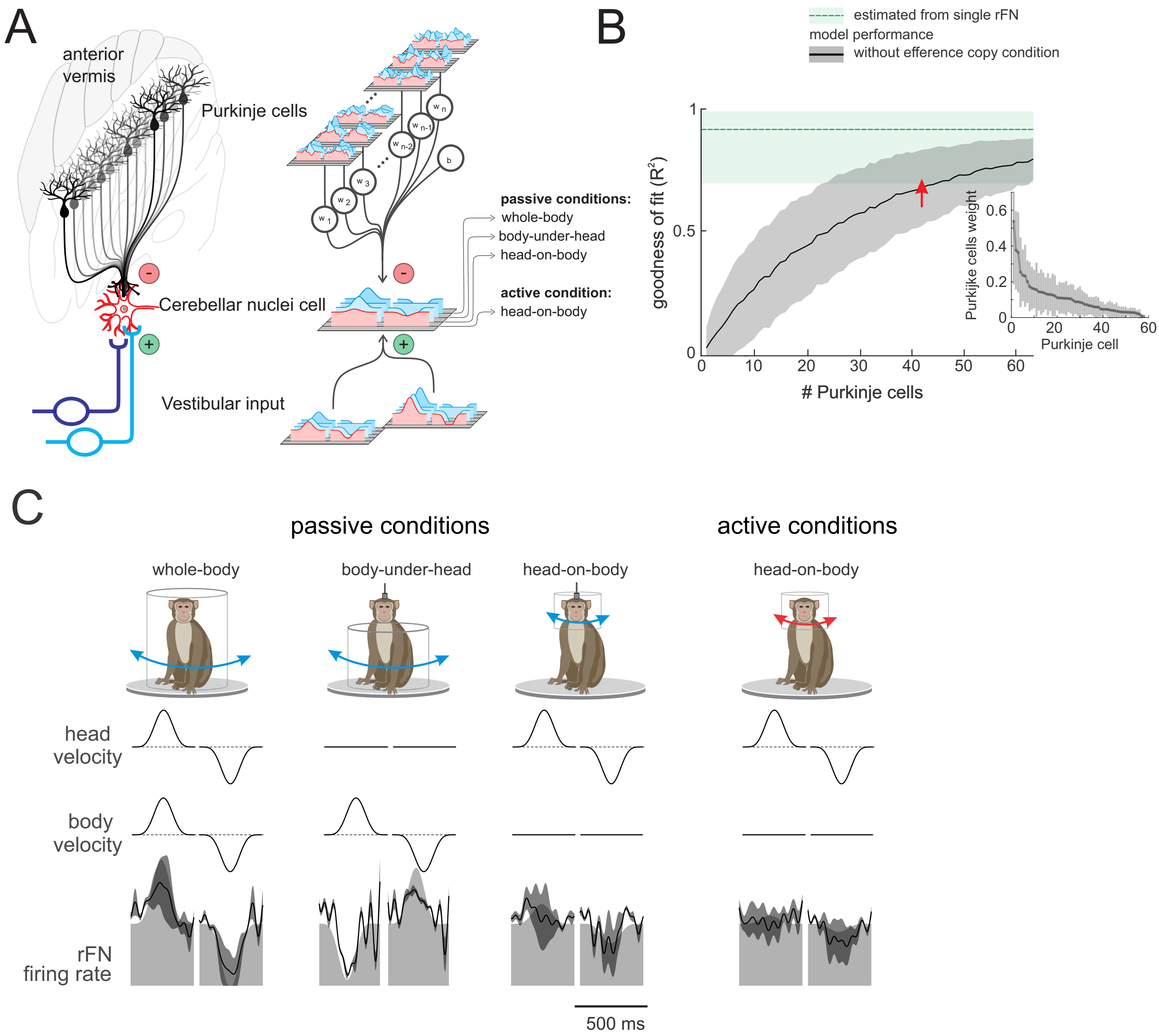

Supplementary Fig. 14 - A simple linear population model of Purkinje cell integration can explain the responses of target bimodal neurons in deep cerebellar nuclei across all self-motion conditions. (A) Left: Illustration of the convergence of multiple Purkinje cells onto a single neuron in the rostral fastigial nucleus (rFN), with different shades representing theoretical differences in the weighing of each Purkinje cell's synapse with the target rFN neuron. Right: Schematic of the linear summation population model used to estimate the firing rate of a target neuron in the rFN. Each Purkinje cell's weight was optimized to generate the best estimate of the average bimodal rFN neuron across conditions (Brooks and Cullen 2013). (B) Model performance as a function of the number of Purkinje cells. The black curve corresponds to the model fit to the simple spike firing rates of all 63 Purkinje cells recorded during our three dynamic conditions (i.e., whole-body, body-under-head, and head-on-body passive movements and active head-on-body movements). The orange curve corresponds to the model fit to simple spike firing rates of all 63 Purkinje cells during these same four conditions as well as simulated responses of these cells recorded in the attempted head movement condition (Figure 5). The variability estimated from a population of rFN bimodal neurons previously described by Brooks and Cullen (2009, 2013) is represented by the green shaded band. Inset: the distribution of computed weights for each Purkinje cell modeled during our three dynamic conditions with 40 Purkinje cells, sorted based on average weight. Data presented as mean values +/- 95% CI. (C) Estimated model firing rates based on a population of 40 Purkinje cells superimposed on the actual average firing rate of a bimodal rostral fastigial nucleus (rFN) neuron (grey shaded region). Solid black lines versus dashed orange lines illustrate firing rate estimations from models that included (i) the four head/body rotation conditions (left) versus (ii) the four conditions as well as the attempted head movement condition (right).
